# Catalytic Oxidation of Benzene over Atomic Active Site AgNi/BCN Catalysts at Room Temperature

**DOI:** 10.3390/molecules29071463

**Published:** 2024-03-25

**Authors:** Xin Zuo, Lisheng Zhang, Ge Gao, Changchun Xin, Bingfeng Fu, Shejiang Liu, Hui Ding

**Affiliations:** 1 School of Environmental Science and Engineering, Tianjin University, Tianjin 300350, China; zuoxin@tju.edu.cn (X.Z.); lishengzhang68@163.com (L.Z.); gaogechn@163.com (G.G.); xinmailbox2023@163.com (C.X.); liushejiang@tju.edu.cn (S.L.); 2 North China Municipal Engineering Design & Research Institute Co., Ltd., Tianjin 300074, China; 3 Shenzhen Yuanqi Environmental Energy Technology Co., Ltd., Futian District, Shenzhen 518045, China; bingfeng_fu@zhifengjm.com

**Keywords:** benzene, AgNi/BCN catalyst, room temperature catalytic oxidation, hydroxyl radical

## Abstract

Benzene is the typical volatile organic compound (VOC) of indoor and outdoor air pollution, which harms human health and the environment. Due to the stability of their aromatic structure, the catalytic oxidation of benzene rings in an environment without an external energy input is difficult. In this study, the efficient degradation of benzene at room temperature was achieved by constructing Ag and Ni bimetallic active site catalysts (AgNi/BCN) supported on boron–carbon–nitrogen aerogel. The atomic-scale Ag and Ni are uniformly dispersed on the catalyst surface and form Ag/Ni-C/N bonds with C and N, which were conducive to the catalytic oxidation of benzene at room temperature. Further catalytic reaction mechanisms indicate that benzene reacted with ·OH to produce R·, which reacted with O_2_ to regenerate ·OH. Under the strong oxidation of ·OH, benzene was oxidized to form alcohols, carboxylic acids, and eventually CO_2_ and H_2_O. This study not only significantly reduces the energy consumption of VOC catalytic oxidation, but also improves the safety of VOC treatment, providing new ideas for the low energy consumption and green development of VOC treatment.

## 1. Introduction

Volatile organic compounds (VOCs) can be converted into pollutants such as O_3_, PM2.5, and photochemical smog after a series of chemical reactions, and are harmful to human health and the environment [1,2]. Benzene, as a typical VOC, mainly comes from the petroleum, printing, textile and dyeing, and rubber industries. It has substantial toxicity, including teratogenicity and carcinogenicity [3,4]. It is important to develop effective treatment methods for the degradation of benzene. At present, the treatment methods for benzene include catalytic oxidation, absorption, membrane separation, incineration, biodegradation, and low-temperature plasma [5]. Catalytic oxidation is considered one of the most promising methods for degrading benzene due to its low energy consumption, high removal efficiency, and low harmful by-products. However, the stable electron-conjugated structure of the benzene (π-π conjugation) increases the difficulty of treatment. Catalytic oxidation of the benzene is considered extremely difficult under ambient temperature and pressure conditions without additional energy such as light, electricity, and heat [6]. Therefore, it is crucial to develop a catalyst that can catalyze benzene without the high-energy environment.

Catalytic oxidation reduces energy consumption and improves the treatment efficiency of VOCs by constructing highly active and stable catalysts [7]. Transition metals, especially precious metal-supported catalysts, exhibit high activity and excellent performance in the oxidation of VOCs [8,9]. The catalytic oxidation temperature of Pt-based or Pd-based catalysts is about 100 K lower than that of other transition metals [9,10,11]. When the Pt particle size is 1.2 nm, the Pt/Al_2_O_3_ catalyst can completely convert benzene to H_2_O and CO_2_ at 145 °C and exhibits excellent stability in long-term reactions [10]. As a readily available precious metal, Ag is introduced into the catalytic oxidation of benzene because of its strong catalytic activity and economic feasibility. Research has showed that Ag-Ce-BTC-C can degrade 1000 ppm toluene at WHSV = 30,000 mL/(g·h) and T_90_ = 226 °C [12]. The Ag/Co_3_O_4_ catalyst containing 2% Ag has more abundant lattice defects, surface oxygen vacancies, and highly reactive oxygen species, contributing to the efficient catalytic oxidation of benzene [13].

Among the metal-supported catalysts, single-atom catalysts (SACS) can realize the efficient oxidation of VOCs under the condition of low metal loading [14]. However, these atoms easily aggregate with the increase in surface free energy, reducing the number of effective catalytic active sites [15]. The “bimetallic atomic active site catalyst” can be constructed by introducing a second metal. The synergistic effect of active centers of two metal atoms can improve the catalytic activity and change the selectivity, effectively overcoming the application restrictions of SACs [16,17]. The bimetallic atomic structure can be confirmed by AC-HAADF-STEM, EXAFS, and first principles simulation (DFT) [18,19,20]. It has been successfully applied in the field of electrochemical reactions, including the oxygen reduction reaction (ORR), CO_2_ reduction reaction (CO_2_RR), N_2_ reduction reaction (NRR), oxygen evolution reaction (OER), and hydrogen evolution reaction (HER) [18,21,22,23,24,25,26]. For example, the catalytic activity of a nitrogen-doped carbon (NC) catalyst (PtFeNC) loaded with isolated Pt and Fe atoms is better than that of Pt/C. DFT calculation results show that a Pt-Fe bimetallic single-atom site accelerates the breaking of O_2_ and the generation of OOH*, which improves the catalytic activity [27].

The oxidation of VOCs can proceed spontaneously at room temperature because of the negative Gibbs free energy change (ΔG < 0). A Na−Pt/AC−R catalyst can achieve 100% degradation of 150 ppm formaldehyde at 25 °C and GHSV = 80,000 h^−1^. The addition of Na can increase the dispersion of Pt on AC, generate more hydroxyl radicals, and directly oxidize HCHO to CO_2_ and H_2_O [28]. Pt/FeOx-400 catalyst can activate O_3_ and H_2_O to produce hydroxyl radicals and completely degrade methanol to CO_2_ and H_2_O at 30 °C [29]. However, the bimetallic active site catalysts have not been applied to the catalytic oxidation of benzene at room temperature because of the low reaction rate of catalytic oxidation of benzene. Therefore, the application of bimetallic atomic catalysts in the catalytic oxidation of benzene at room temperature needs to be further studied. 

In this study, the bimetallic atomic active sites were anchored on the surface of BCN aerogels by the impregnation method. Benzene was used as a probe to evaluate the catalyst. The specific surface area, pore volume, and crystal structure of the catalyst were characterized by BET and XRD. The microstructure of the catalyst was studied by SEM, TEM, EDS, and AC-HADDF-STEM; FTIR and XPS were used to study the surface characteristics and elemental chemical state of the catalyst, and further explore the mechanism of catalytic oxidation of benzene at room temperature.

## 2. Results and Discussion

### 2.1. Catalytic Oxidation of Benzene at Room Temperature

**Figure 1:**
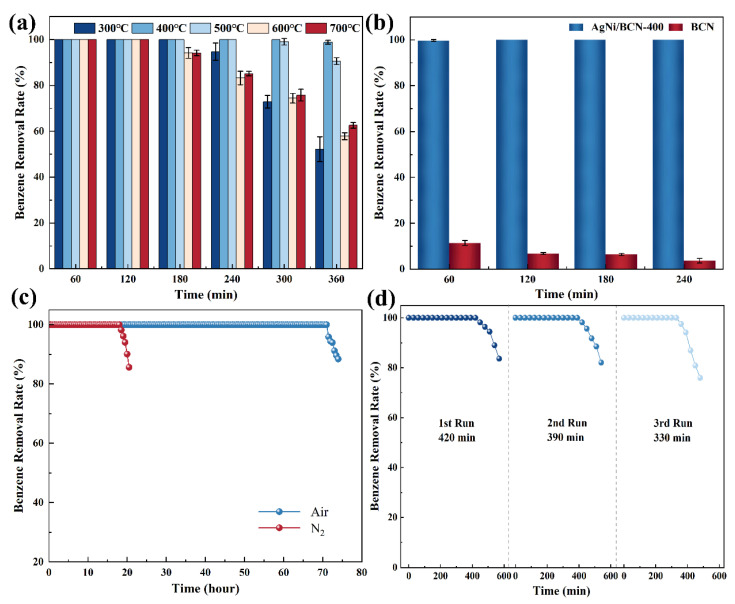
Performance of catalysts for benzene oxidation: (**a**) effect of calcination temperature on the catalytic performance of benzene; (**b**) comparison experiment with carrier; (**c**) long-term stability; (**d**) cyclic stability.

The catalyst evaluation experiments were carried out at 25 °C. In order to select the optimal catalyst, catalysts with different calcination temperatures were used to oxidize 1400 mg/m^3^ benzene. As shown in Figure 1a, the catalyst calcined at 400 °C performed best, capable of removing 98% benzene in 6 h. As shown in Figure 1b, the BCN support without metal loading reached adsorption saturation at the beginning, and the benzene removal rate dropped below 5% after 4 h, indicating that the loading of Ag and Ni enhanced the catalytic activity of the catalyst. 

In order to test the stability of the catalyst, long-term stability and recycling experiments were carried out. As shown in Figure 1c, benzene conversion remained 100% after 71 h in air atmosphere. However, in N_2_ atmosphere, the catalyst only adsorbed benzene and reached adsorption saturation at 18 h. This indicates that O_2_ and H_2_O in the air may participate in the catalytic oxidation of benzene as reactants. In the cycle test, the used catalyst was regenerated by calcination at 400 °C (N_2_ atmosphere, heating rate = 5 °C/min) for 2 h and tested under the same conditions. After three cycles, the conversion rate of benzene remained 100% at 330 min (Figure 1d). Therefore, the AgNi/BCN-400 catalyst has good stability, and using a calcining catalyst at 400 °C for 2 h is an effective catalyst regeneration method.

### 2.2. AgNi/BCN Catalyst Bulk Structure

To explore the effect of specific surface area and pore volume on the catalytic oxidation performance of catalysts, BET was used to carry out N_2_ physical adsorption–desorption experiments on the metal-free BCN aerogel carrier, and AgNi/BCN-300, AgNi/BCN-400, AgNi/BCN-500, AgNi/BCN-600, and AgNi/BCN-700 catalysts. As shown in Figure S1, the adsorption curve and desorption curve of the isotherm of the sample are inconsistent. There is a H4-type hysteresis loop in the IUPAC classification and it is without an obvious adsorption saturation platform. This shows that micropores and mesopores exist simultaneously in the catalyst sample, which has an irregular porous structure. The pore size of the catalyst is 2–4 nm, which is more suitable for the loading and uniform dispersion of metal. As shown in Table 1, it can be seen that the specific surface area and pore volume of the catalyst loaded with Ag and Ni species increased significantly, and the average pore size decreased. This is because the carrier was calcined again after loading the metal species, and the N element is lost in the form of ammonia gas at high temperature, resulting in more pores.

**Figure 2:**
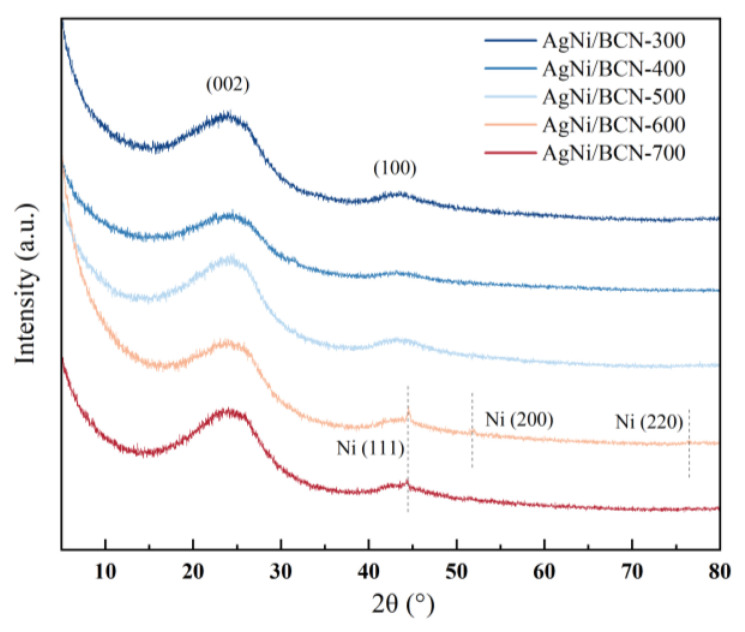
XRD analysis of AgNi/BCN-X catalysts.

As shown in Figure 2, XRD was used to characterize the catalyst at different calcination temperatures. The catalysts have two wide crystal plane diffraction peaks around 25.4° and 43.6°, corresponding to (002) and (101) crystal planes of graphite carbon, respectively [30,31]. The low intensity of the two broad peaks indicates that the crystallinity of the BCN aerogel carrier is low. When the calcination temperature is 600 °C, there are three diffraction peaks at 44.5°, 51.9°, and 76.5° belonging to (111), (200), and (220) crystal planes of Ni^0^ [32,33,34]. This indicates that Ni successfully loaded.

When the calcination temperature is 700 °C, the diffraction peak at 44.6° is the (111) crystal plane of Ni^0^. This indicates that when the calcination temperature was more significant than 600 °C, there were nickel nanoparticles (Ni NPs) in the AgNi/BCN-X catalyst. Because of the high-temperature annealing in the inert gas atmosphere, the Ni^n+^ (n = 2, 3) in the catalyst was reduced to form Ni^0^ [35]. When the calcination temperature is 300 °C, 400 °C, or 500 °C, there is no obvious diffraction peak of Ni, indicating that the crystallinity of Ni is low. The AgNi/BCN catalysts prepared at different calcination temperatures have no crystal diffraction peak of Ag^0^, indicating that the catalysts at the five calcination temperatures have no large Ag nanoparticles because the crystallinity of Ag on the catalyst surface is low.

### 2.3. Morphology of AgNi/BCN Catalyst

The BCN aerogel and AgNi/BCN-400 catalysts were analyzed by SEM, TEM, and EDS surface scanning to determine the micromorphology, particle size, crystal phase structure, and element distribution of the catalyst. As shown in Figure S2, the BCN aerogel without metal species showed a spongy porous structure with a high specific surface area of the catalyst. This was conducive to the stable loading of Ag and Ni active atoms and the adsorption of reactant molecules by the catalyst. As shown in Figure S3, compared with the BCN carrier, AgNi/BCN-400 has more dense pores with a smaller volume, which is consistent with the specific surface area and average pore size results in the BET results.

**Figure 3:**
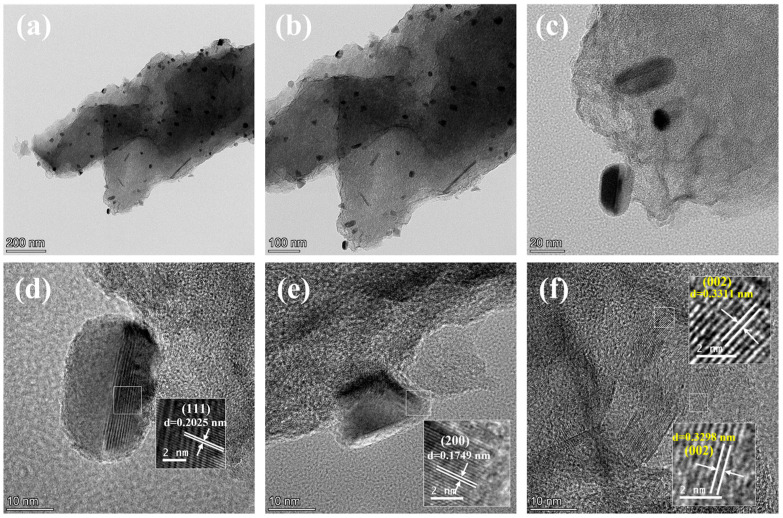
HRTEM images of AgNi/BCN-400 catalyst under different scales: (**a**) 200 nm; (**b**) 100 nm; (**c**) 20 nm; (**d**) 10 nm, Ni NPs; (**e**) 10 nm, Ni NPs; (**f**) 10 nm, Graphite carbon structure.

The pores of the catalyst are uniformly loaded with spherical and rod-shaped nanostructures, which may be Ni NPs. As shown in Figure 3a–c, the catalyst has a translucent nanosheet structure. Some spherical and rod-shaped nanoparticles are distributed on the nanoflake carrier, and the distribution of nanoparticles is relatively uniform. In Figure 3d, the lattice stripe of d = 0.2025 nm corresponding to the (111) plane of Ni can be measured. In Figure 3e, the lattice stripe of d = 0.1749 nm can be measured, which corresponds to the (200) plane of Ni [36,37,38]. The existence of Ni NPs was further confirmed, and the diameter of Ni NPs was small (about 20 nm) without aggregation. This also explains why there was no obvious crystal plane diffraction peak of Ni^0^ in the XRD spectrum of the AgNi/BCN-400 catalyst. In Figure 3f, the lattice stripes with d = 0.3311 nm and d = 0.3298 nm, which belong to the (002) crystal plane of graphite carbon, can be measured. This is consistent with the diffraction peak results of the (002) crystal plane of graphite carbon in XRD.

**Figure 4:**
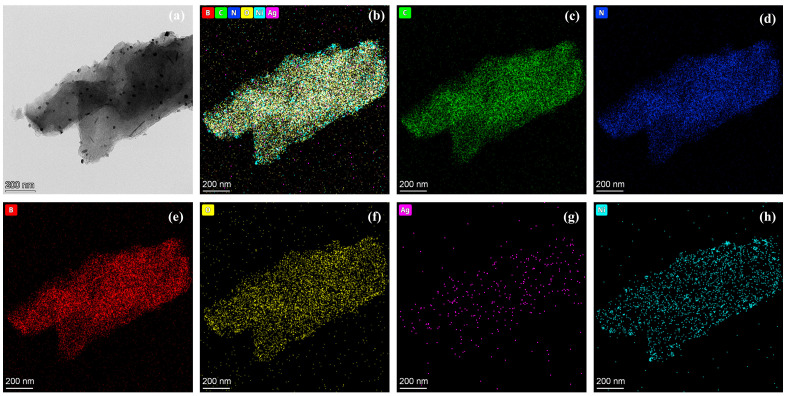
HADDF-STEM images and EDS mapping images of AgNi/BCN-400 catalyst: (**a**) TEM (**b**) EDS (**c**) EDS of C; (**d**) EDS of N; (**e**) EDS of B; (**f**) EDS of O; (**g**) EDS of Ag; (**h**) EDS of Ni.

As shown in Figure 4, the catalyst surface contains elements C, N, B, O, Ag, and Ni, and each element is evenly distributed. Ni has a small range of aggregation compared to other elements, which corresponds to the white nanoparticles in Figure 4a. This indicates the formation of Ni NPs, which is consistent with the results of (111) and (200) crystal planes of Ni in TEM. The higher dispersion of Ag and no diffraction peak of Ag^0^ suggest that Ag may exist in monatomic form. In addition, the Ag and Ni elements uniformly distributed on the support surface further confirmed that the two metals were successfully loaded on the BCN aerogel.

**Figure 5:**
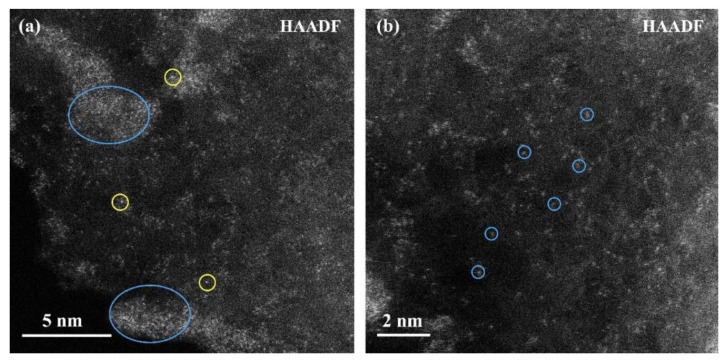
AC-HADDF-STEM images of AgNi/BCN-400 catalyst: (**a**) Ni NPs and Ag single atoms, (**b**) Ni single atoms.

As shown in Figure 5a, most areas on the surface of the catalyst are scattered single atoms and atomic clusters, and a small area forms larger clusters (blue ellipse). Combined with the HRTEM and HADDF-STEM results at the scale of 200 nm of the catalyst, this is because of the nanoparticles of Ni. In addition, it can be seen that there are single atoms with high brightness in the mono-atomic dispersion area. The large atomic number produces high brightness. Therefore, these brighter atoms are Ag mono-atoms.

There are also some Ni single atoms around Ag single atoms (yellow circle), indicating that there may be interactions between Ag and Ni single atoms to aggregate and form small atomic clusters. It can be seen from Figure 5b that a large number of Ni single atoms are distributed on the surface of the catalyst, which further indicates that there are three modes of Ni in the AgNi/BCN-400 catalyst: (1) Ni single atoms are dispersed on the surface of the catalyst alone; (2) it exists as Ni NPs; and (3) the interaction between a Ni single atom and a Ag single atom causes it to surround the Ag single atom.

### 2.4. Coordination Environment and Valence State of AgNi/BCN Catalyst

**Figure 6:**
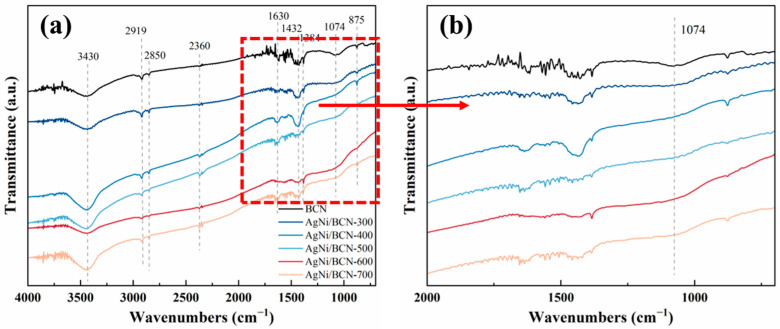
FTIR spectra of AgNi/BCN-X catalysts: (**a**) full spectrum; (**b**) detail amplification.

As shown in Figure 6, the adsorbed substances and functional groups on the surface of the AgNi/BCN-X were studied by FTIR. The peaks at wave numbers 3430 cm^−1^ and 1630 cm^−1^ are attributed to the stretching vibration of surface hydroxyl groups (-OH) and the bending vibration of adsorbed water molecules, respectively [39,40]. The peak at 1432 cm^−1^ is attributed to the bending vibration of -OH in -COOH [41]. Compared with other catalysts, AgNi/BCN-400, which has the best catalytic performance, has larger characteristic peak areas at 3430 cm^−1^, 1630 cm^−1^, and 1432 cm^−1^, indicating that there are more hydroxyl and other oxygen-containing groups on the surface of AgNi/BCN-400 [40]. The hydroxyl and other oxygen-containing groups may participate in the catalytic oxidation reaction, which is conducive to the catalytic degradation of benzene at room temperature. It has been shown that the surface defects caused by Ni loading are beneficial to the adsorption of hydroxyl and water molecules on the surface [40]. In addition, hydroxyl can also enhance the interaction between the metal and support [42]. The characteristic peak at 1074 cm^−1^ corresponds to the stretching vibration of C-N [32,43]. It indicates that N successfully dopes into carbon to form a C-N structure.

**Figure 7:**
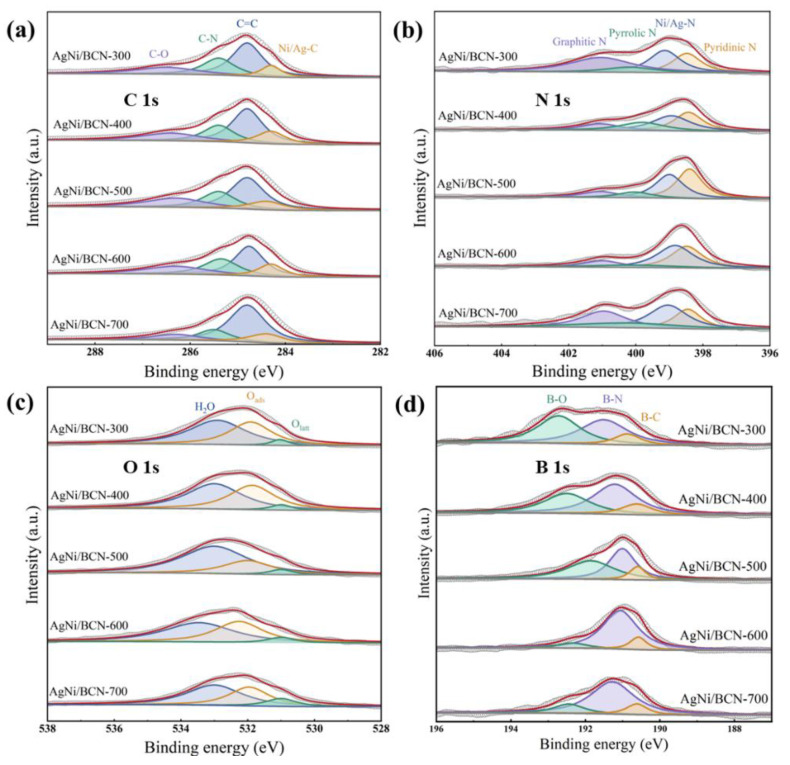
The high resolution XPS spectra images of AgNi/BCN-X catalysts: (**a**) the high-resolution C 1s XPS spectra images; (**b**) the high-resolution N 1s XPS spectra images; (**c**) the high-resolution O 1s XPS spectra images; (**d**) the high-resolution B 1s XPS spectra images.

As shown in Figure 7, the chemical valence of the constituent elements and the molecular bonds of the AgNi/BCN-X were studied by XPS. After conducting a full scan of the AgNi/BCN-X catalyst using XPS, it was found that the catalysts prepared at different calcination temperatures exhibited characteristic photoelectron peaks of C 1s, N 1s, O 1s, B 1s, Ni 2p, Ag 3d, and Na 1s in the XPS spectra (Figure S4). As shown in Figure 7a, the C 1s photoelectron peaks of catalysts prepared at different calcination temperatures can be convoluted into four peaks. The peak of binding energy at 284.3–284.4 eV belongs to the metal–carbon bond (Ni/Ag-C) [38]. The peak of 284.8 eV was identified as a sp^2^ hybrid graphite (C=C) structure [44]. The peak of 285.4–285.5 eV is the C-N bond corresponding to the C-N bond in FTIR [36,45]. The peak of 286.3–286.5 eV belongs to the C-O bond [46,47]. The peak of Ni/Ag-C appeared in the C 1s spectrum of the catalyst, indicating that Ag and Ni species in AgNi/BCN formed a coordination with C. This further confirmed that Ag and Ni interacted with the support and were successfully loaded on the support. AgNi/BCN-400, which had the best catalytic performance, contained the most Ni/Ag-C, indicating that the catalytic oxidation of benzene at room temperature was related to Ni/Ag-C in the AgNi/BCN catalyst. The C-N bond appeared in the C 1s spectrum, indicating that the N of urea successfully interacted with the C in the raw material to form a stable BCN aerogel carrier. In addition, the content of the C-N bond decreased with the increase in calcination temperature, which may be due to the loss of N in the state of ammonia. As shown in Figure 7b, the N1s photoelectron peaks of catalysts prepared at different calcination temperatures can be convoluted into four peaks. The peak of binding energy at 398.3–398.5 eV was pyridinic N, the peak near 399.0 eV was metal nitrogen (Ni/Ag-C), the peak near 400.0 eV was pyrrolic N, and the peak near 401.0 eV was graphite N [48]. A peak of the Ni/Ag-C bond appears in the N 1s spectrum, indicating that Ag and Ni species are coordinated with N, and 399.0 eV was the Ni-N bond. From the characteristic peaks of pyridine nitrogen and pyrrole nitrogen in the N 1s spectrum, we can infer the diversity of bonding modes between N and C. AgNi/BCN-400 contains the highest pyrrole nitrogen ratio, indicating that the catalytic oxidation activity of the catalyst for benzene at room temperature may be related to pyrrole nitrogen. Pyrrole nitrogen, which has a unique electronic structure, can effectively promote the adsorption of reactant molecules [49,50]. 

As shown in Figure 7c, the O 1s photoelectron peaks of catalysts prepared at different calcination temperatures can be convoluted into three peaks. The peak at 531.0 eV is lattice oxygen (O_latt_), the peak at 531.8–532.3 eV is adsorbed oxygen (O_ads_), and the peak at 533.0–533.5 eV is adsorbed water [51]. The catalytic oxidation activity of the catalyst for benzene is related to the surface oxygen species. With the increase in calcination temperature, the overall trend of adsorbed oxygen/lattice oxygen increases first and then decreases. As shown in Table S2, AgNi/BCN-400 has the highest ratio of adsorbed oxygen/lattice oxygen, consistent with the result that AgNi/BCN-400 has the most oxygen-containing groups in FTIR. It may be that adsorbed oxygen more easily participates in the catalytic oxidation reaction of benzene, and the adsorption of oxygen on the surface is beneficial to the catalytic oxidation of benzene at room temperature [52,53]. As shown in Figure 7d, the B 1s photoelectron peak of the AgNi/BCN-X catalyst can be convoluted into three peaks: the peak of 190.5–190.8 eV is the B-C bond, the peak of 191.0–191.4 eV is the B-N bond, and the peak near 533.0–533.5 eV is the B-O bond [54,55]. The three bonds indicate that B has been well doped into the C and N frameworks to form a stable BCN aerogel structure. As shown in Table S3, the AgNi/BCN-400 catalyst with the best catalytic performance has the highest proportion of B-C bonds, indicating that the doping of B may affect the catalytic performance of the catalyst. It was found that the doping of B in CNTs can change the Ni 3D orbital, optimize the three-dimensional electron occupancy of the Ni atom, and improve the OER electrocatalytic activity of the catalyst [56].

**Figure 8:**
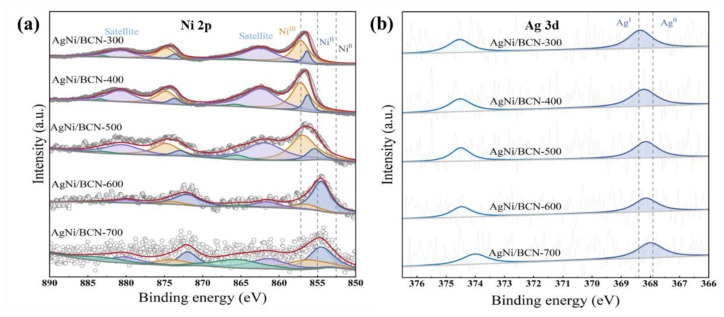
The high-resolution XPS spectra images of AgNi/BCN-X catalysts: (**a**) the high-resolution Ni 2p XPS spectra images; (**b**) the high-resolution Ag 3d XPS spectra images.

As shown in Figure 8a, the binding energies of the photoelectron peaks of Ni^0^ are located at 852.5 eV (2p_3/2_) and 869.6 eV (2p_1/2_). The characteristic electron peaks of Ni^2+^ are located at 855.0 eV (2p_3/2_) and 873.7 eV (2p_1/2_). The characteristic peaks attributed to Ni^3+^ are located at 857.0 eV (2P_3/2_) and 875 eV (2p_1/2_) [46,57]. The Ni on the surface of the catalyst calcined at 300–500 °C is Ni^2+^ and Ni^3+^. According to the high-resolution XPS spectra of C 1s and N 1s, Ni mainly forms coordination with C and N, resulting in the formation of Ni^2+^ and Ni^3+^. Due to the high ratio of Ni^2+^ and Ni^3+^, Ni mainly exists on the AgNi/BCN-400 surface as a single atom, which is consistent with the results of HADDF-STEM. The two characteristic electronic peaks of the catalysts calcined at 600 °C and 700 °C were between 852.5–855.0 eV and 855.0–857.0 eV, indicating the coexistence of Ni^0^, Ni^2+^ and Ni^3+^ on the surface of the catalysts calcined at these temperatures. The increase in Ni^0^ indicates that the surface of the catalyst is reduced to Ni^0^ under high-temperature calcination. As shown in Figure 8b, the characteristic peaks with binding energies at 368.4 eV (3d_5/2_) and 374.5 eV (3d_3/2_) belong to Ag^1+^ [58]. The 367.9 eV (3d_5/2_) and 374.0 eV (3d_3/2_) photoelectron peaks are Ag^0^ [59,60,61]. Ag on the surface of the catalyst calcined at 300 °C mainly exists in the form of Ag^1+^. With the increase in calcination temperature, the photoelectron peak of XPS tends to move to the right, indicating that a part of Ag^1+^ is reduced to Ag^0^, and the Ag on the surface of AgNi/BCN-400 is between Ag^1+^ and Ag^0^. Combined with the XPS results, this may be caused by the formation of coordination between Ag, and C and N. The results of TEM and HADDF-STEM showed that Ag atoms were distributed on the catalyst’s surface, which was consistent with the result that there was no diffraction peak of Ag^0^ in XRD.

### 2.5. The Catalytic Mechanism of AgNi/BCN-400

**Figure 9:**
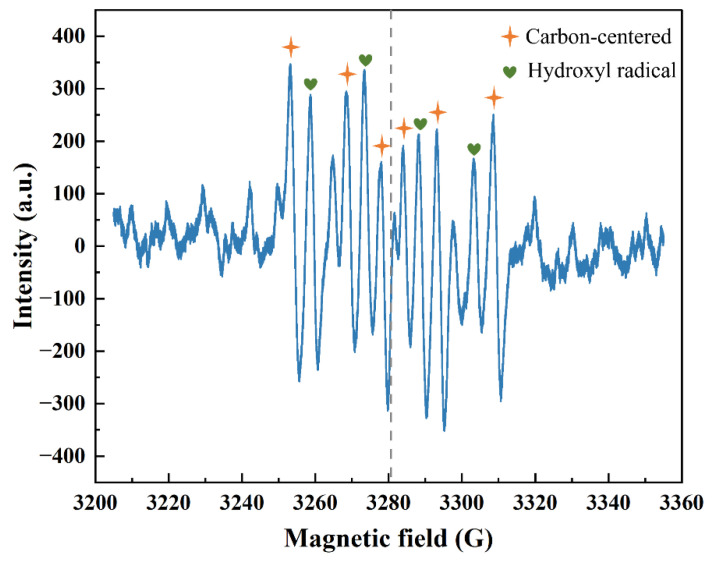
EPR spectrum of radical species.

To explore the reactive oxygen species that play a role in the reaction and clarify the mechanism of benzene degradation at room temperature, EPR was used to detect the reactive oxygen species. In addition, FTIR was used to explore the surface species of the AgNi/BCN-400 catalyst before and after the reaction. As shown in Figure 9, six peaks (orange quadrangular star) represent the spin adduct (DMPO-R, A_Hβ_~24–28 G, A_N_~15–17 G) of the alkenyl carbon center radical (R·). A_Hβ_ was 24.66 G, and A_N_ was 15.30 G, 15.57 G, 15.23 G, and 15.43 G, respectively. The relative intensities of the characteristic peak of hydroxyl radical spin adduct (DMPO-OH) were close to 1:2:2:1 (green heart); A_Hβ_ was 14.77 G, 14.70 G, and 14.95 G, and A_N_ was 14.17 G and 15.47 G. This indicates that the oxygen and H_2_O in the air are activated by the catalyst to form ·OH reactive oxygen species, which are the key free radicals for benzene ring opening at room temperature [29,62]. The existence of R· also indicates that smaller olefin groups are formed after benzene ring opening. The ·OH generated during the oxidation of VOCs could attack the C-H bond to extract H atoms or add ·OH to unsaturated sites, resulting in R· [63]. Although the oxidation activity of R· is relatively weak, R· can combine with O_2_ to form alkyl peroxy radicals (ROO·) [64]. ROO· generates a carbon-centered intermediate of hydroperoxyl alkyl radicals (·QOOH) through an internal hydrogen shift, but this is difficult to observe, and it will degrade and decay rapidly to generate ·OH [65]. This also explains why R· and ·OH are detected simultaneously in the catalytic oxidation of benzene at room temperature.

**Figure 10:**
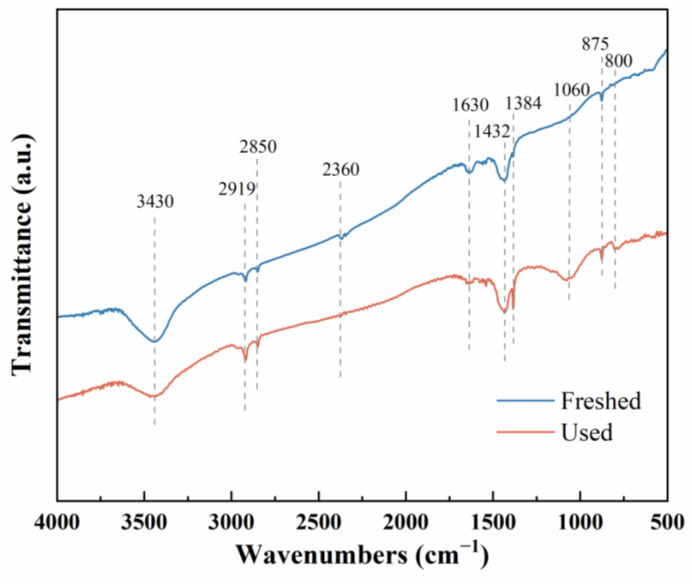
FTIR spectra of the AgNi/BCN-400 catalyst before and after the reaction.

As shown in Figure 10, the infrared characteristic peaks at wave numbers 3430 cm^−1^ and 1630 cm^−1^ are the stretching vibration of the surface hydroxyl group (-OH) and the bending vibration of the adsorbed water molecules, respectively. After the reaction, the surface hydroxyl of the catalyst is significantly reduced, indicating that the surface hydroxyl participates in the catalytic oxidation of benzene at room temperature. This is consistent with the highest proportion of oxygen adsorbed on the AgNi/BCN-400 surface with the XPS analysis. The characteristic peak at 1432 cm^−1^ was confirmed as the bending vibration of -OH in -COOH. The catalyst peak area increased, indicating the benzene ring-opening reaction and that the formation of small molecular carboxylic acids occurred. In addition, the stretching vibration peak of the C-O bond in 1384 cm^−1^ carbonate was significantly enhanced, which showed that a large amount of CO_2_ was generated and deposited on the catalyst surface. The intensities of characteristic peaks at 2919 cm^−1^ and 2850 cm^−1^ increased, which were attributed to the asymmetric and symmetric stretching vibrations of methyl and methylene C-H in aromatic hydrocarbons. It can be seen that benzene was adsorbed on the catalyst surface during the reaction. The enhancement of the characteristic peaks (875 cm^−1^ and 800 cm^−1^) corresponding to the out-of-plane bending vibration of the benzene ring C-H further confirms this view. The 1060 cm^−1^ peak is the stretching vibration of the C-O bond of alcohol, and its increased strength indicates that alcohols are generated in the catalytic oxidation of benzene at room temperature [52,66,67]. This indicates that benzene will be oxidized under the strong oxidation of ·OH to form alcohols, carboxylic acids, and, ultimately, CO_2_ and H_2_O.

## 3. Experimental

### 3.1. Chemicals and Materials

In this study, boron–carbon–nitrogen (BCN) aerogel was used as the catalyst carrier. It combines the advantages of carbon materials and hexagonal boron nitride with adjustable porosity and large surface area, which can improve the ability to adsorb reactants and is more conducive to loading metal species. All the rest of the chemicals were analytical grade and used as received without further purification (Table S1).

### 3.2. Catalyst Preparation

Ag and Ni were loaded on BCN aerogel using the impregnation method. H_3_BO_3_ (0.5 g), CO (NH_2_)_2_ (2 g), soluble starch (5 g), and NaCl (5 g) were dissolved in deionized water. Ultrasound treatment was performed during this period. The hydrogel precursor was formed after being placed in air at room temperature for 4 h and put into a tubular furnace. It was calcined at 800 °C in a nitrogen atmosphere for 4 h, and the heating rate was five °C/min. AgNO_3_ (1.86 mL, 0.05 mol/L) and Ni (NO_3_)_2_·6H_2_O (3.70 mL, 0.05 mol/L) solutions were put into 200 mL deionized water. The 6 g BCN aerogel carrier was placed in the above clarified mixed solution, and the catalyst was uniformly dispersed by ultrasound for 30 min. The metal in the precursor solution was loaded on the BCN carrier by magnetic stirring (600 rpm) at 60 °C for 4 h. The product was washed three times with deionized water and dried in an oven at 60 °C for 12 h. The samples were calcined in a nitrogen atmosphere at different temperatures (300 °C, 400 °C, 500 °C, 600 °C, and 700 °C) for 180 min to obtain the AgNi/BCN-X catalyst (X is the calcination temperature).

### 3.3. Catalyst Evaluation

The catalytic activity of the catalyst was evaluated by a self-assembled VOC catalyst evaluation device and the online gas chromatography detection method (GC-6890A, Lunan Analytical Instrument Co., Ltd., Tengzhou, China). The hydrogen and air generators were used to provide the flame combustion gas and booster gas in the hydrogen flame ionization detector (FID, Lunan Analytical Instrument Co., Ltd., China) of the gas chromatograph. The temperature of benzene vapor generation was controlled at 1–5 °C by a circulating refrigerator. The reaction temperature of the fixed bed reactor was controlled at 25 °C. The inlet concentration of benzene (*C_in_*) was adjusted by controlling the airflow, N_2_ flow of purging benzene, and benzene generation temperature. Weight hourly space velocity (WHSV) was controlled by controlling airflow and catalyst filling quality. The relative humidity (RH) of the reaction was adjusted by controlling the N_2_ flow into the water vapor bubbling bottle and the amount of desiccant in the drying tube, and the RH was monitored in real time with a hygrometer. The gas after reaction was divided into two routes: one entered the gas chromatograph to monitor the outlet concentration of benzene (*C_out_*), and the other was discharged. The benzene removal rate (*ω*) was calculated by Formula (1):

(1) $$\omega =\frac{C_{in}-C_{out}}{C_{in}}\times 100\%$$

where “*ω*” is the benzene removal rate, %; *C_in_* is the benzene inlet concentration, mg/m^3^; and *C_out_* is the benzene outlet concentration, mg/m^3^.

### 3.4. Catalyst Characterization

To explore the relationship between catalytic oxidation performance and specific surface area and pore volume, Brunauer–Emmett–Teller (BET) theory was used to determine the specific surface area and pore size of the catalyst. An X-ray diffractometer (XRD, Rigaku Ultima IV, Rigaku, Tokyo, Japan) was used to analyze the crystal structure of the catalysts and the loading form of Ag and Ni species on the catalyst support. Scanning electron microscopy (SEM, Scientific Apreo 2C, Thermo, Waltham, MA, USA) and transmission electron microscopy (TEM, Talos F200S G2, Thermo, Waltham, MA, USA) were used to observe the microstructure, particle size, crystal structure, and element distribution of the samples. Energy-dispersive X-ray spectroscopy (EDS) was used to explore the element distribution of the sample. A Fourier transform infrared spectrometer (FTIR, FTIR-650, Tianjin Port East Science and Technology Development Co., Ltd., Tianjin, China) was used for qualitative analysis of functional groups in samples. An Aberration-Corrected High-Angle Annular Dark-Field Scanning Transmission Electron Microscope (AC-HADDF-STEM, Titan Themis 60-300, FEI, Eindhoven, The Netherlands) was used to observe the existence of single atoms. X-ray photoelectron spectroscopy (XPS, Fisher ESCALAB 250Xi, Thermo, Waltham, MA, USA) was used to explore the chemical composition and elemental valence of the sample surface. An electron paramagnetic resonance spectrometer (EPR, JEOL JES-FA200, JEOL, Tokyo, Japan), which can be used to detect free radicals containing at least one unpaired electron, was used to test the tail gas from catalytic oxidation of benzene at room temperature.

## 4. Conclusions

This study used impregnation to prepare the bimetallic atomic active site catalyst AgNi/BCN for catalytic oxidation of benzene at room temperature. It also explored the structure–activity relationship of AgNi/BCN catalysts for catalytic oxidation of benzene at room temperature through a series of characterization experiments. Additionally, the study involved further exploration of the catalytic oxidation mechanism of benzene. 

The results showed that the AgNi/BCN-400 catalyst had a large specific surface area (503.0 m^2^/g) and total pore volume (0.3394 cm^3^/g). This catalyst has a loose porous structure, with nanosheets and Ni NPs on its surface. Most of the Ag and Ni disperses at the atomic level on the surface of the catalyst and forms Ag/Ni-C/N bonds with C and N. AgNi/BCN-400, which has the best catalytic performance, has the highest Ni/Ag-C content, and a small part of Ni exists in the form of Ni NPs. Therefore, the large specific surface area and total pore volume of the AgNi/BCN-400 catalyst are conducive to the adsorption and further activation of reactant molecules, and the atomic dispersion of Ag and Ni, while Ag/Ni-C/N bonds and surface-adsorbed oxygen species are conducive to the catalytic oxidation of benzene at room temperature. The degradation mechanism can be described as benzene being attacked by ·OH to open the ring to produce R·, and the generated R· further combining with O_2_ and undergoing isomerization to generate ·OH. The FTIR spectra of the catalyst before and after the reaction show that benzene will be oxidized under the strong oxidation of ·OH to form alcohols, carboxylic acids, and, ultimately, CO_2_ and H_2_O.

## Figures and Tables

**Table 1:** BET specific surface area, average pore size, and pore volume of AgNi/BCN catalysts synthesized at different pyrolysis temperatures.

Sample	Specific Surface Area (m^2^/g)	Average Pore Size (nm)	Pore Volume (mL/g)
BCN	61.8	2.96	0.0457
AgNi/BCN-300	545	2.75	0.375
AgNi/BCN-400	503	2.70	0.339
AgNi/BCN-500	405	2.44	0.246
AgNi/BCN-600	555	2.61	0.363
AgNi/BCN-700	498	2.66	0.331

## Data Availability

The raw data supporting the conclusions of this article will be made available by the authors on request.

## Supplementary Materials

The following supporting information can be downloaded at: https://www.mdpi.com/article/10.3390/molecules29071463/s1. Figure S1. N2 adsorption-desorption experiments: (a) N2 adsorption-desorption isotherms; (b) Pore size distribution. Figure S2. SEM images of BCN aerogel carrier. Figure S3. SEM images of AgNi/BCN-400 catalyst. Figure S4. XPS survey scan of AgNi/BCN-X catalysts. Table S1. Experimental agents. Table S2. Relative content of oxygen species in AgNi/BCN-X catalysts. Table S3. Relative content of boron species in AgNi/BCN-X catalysts.

## References

[1] Mu Y., Williams P.T. (2022). Recent advances in the abatement of volatile organic compounds (VOCs) and chlorinated-VOCs by non-thermal plasma technology: A review. Chemosphere.

[2] Chang T., Wang Y., Wang Y., Zhao Z., Shen Z., Huang Y., Veerapandian S.K.P., De Geyter N., Wang C., Chen Q. (2022). A critical review on plasma-catalytic removal of VOCs: Catalyst development, process parameters and synergetic reaction mechanism. Sci. Total Environ..

[3] Zeng J., Liu X., Wang J., Lv H., Zhu T. (2015). Catalytic oxidation of benzene over MnOx/TiO_2_ catalysts and the mechanism study. J. Mol. Catal. A Chem..

[4] Luo Y., Lin D., Zheng Y., Feng X., Chen Q., Zhang K., Wang X., Jiang L. (2020). MnO_2_ nanoparticles encapsuled in spheres of Ce-Mn solid solution: Efficient catalyst and good water tolerance for low-temperature toluene oxidation. Appl. Surf. Sci..

[5] Qin C., Dang X., Huang J., Teng J., Huang X. (2016). Plasma-catalytic oxidation of adsorbed toluene on Ag-Mn/gamma-Al_2_O_3_: Comparison of gas flow-through and gas circulation treatment. Chem. Eng. J..

[6] Ding H., Xue L., Cui J., Wang Y., Zhao D., Zhi X., Liu R., Fu J., Liu S., Fu B. (2023). Catalytic degradation of benzene at room temperature over FeN_4_O_2_ sites embedded in porous carbon. J. Hazard. Mater..

[7] Maleki H., Hüsing N. (2018). Current status, opportunities and challenges in catalytic and photocatalytic applications of aerogels: Environmental protection aspects. Appl. Catal. B Environ..

[8] Kim S.-I., Im M., Cho E., Jang H., Jang S.Y., Kim D.W., Kim K.W., Heo I., Kim Y.J., Lee J.H. (2022). Effects of thermal aging on the electronic and structural properties of Pt-Pd and toluene oxidation activity. Sci. Total Environ..

[9] Peng R., Li S., Sun X., Ren Q., Chen L., Fu M., Wu J., Ye D. (2018). Size effect of Pt nanoparticles on the catalytic oxidation of toluene over Pt/CeO_2_ catalysts. Appl. Catal. B Environ..

[10] Chen Z., Mao J., Zhou R. (2019). Preparation of size-controlled Pt supported on Al_2_O_3_ nanocatalysts for deep catalytic oxidation of benzene at lower temperature. Appl. Surf. Sci..

[11] Liu Y., Dai J., Liu N., Wu Y., Huang J., Zheng Y., Li Q. (2021). Oxygen-enriched biomass-activated carbon supported platinum nanoparticles as an efficient and durable catalyst for oxidation in benzene. ACS Sustain. Chem. Eng..

[12] Wang Y., Bi F., Wang Y., Jia M., Tao X., Jin Y., Zhang X. (2021). MOF-derived CeO_2_ supported Ag catalysts for toluene oxidation: The effect of synthesis method. Mol. Catal..

[13] Ma X., Yu X., Ge M. (2021). Highly efficient catalytic oxidation of benzene over Ag assisted Co_3_O_4_ catalysts. Catal. Today.

[14] Pan Y., Zhang C., Liu Z., Chen C., Li Y. (2020). Structural regulation with atomic-level precision: From single-atomic site to diatomic and atomic interface catalysis. Matter.

[15] Li X., Bi W., Zhang L., Tao S., Chu W., Zhang Q., Luo Y., Wu C., Xie Y. (2016). Single-atom Pt as Co-catalyst for enhanced photocatalytic H_2_ Evolution. Adv. Mater..

[16] Zhu Z., Yin H., Wang Y., Chuang C.-H., Xing L., Dong M., Lu Y.-R., Casillas-Garcia G., Zheng Y., Chen S. (2020). Coexisting single-atomic Fe and Ni sites on hierarchically ordered porous carbon as a highly efficient ORR electrocatalyst. Adv. Mater..

[17] He C., Cheng J., Zhang X., Douthwaite M., Pattisson S., Hao Z. (2019). Recent advances in the catalytic oxidation of volatile organic compounds: A review based on pollutant sorts and sources. Chem. Rev..

[18] Wang J., Xu R., Sun Y., Liu Q., Xia M., Li Y., Gao F., Zhao Y., Tse J.S. (2021). Identifying the Zn–Co binary as a robust bifunctional electrocatalyst in oxygen reduction and evolution reactions via shifting the apexes of the volcano plot. J. Energy Chem..

[19] Tian S., Wang B., Gong W., He Z., Xu Q., Chen W., Zhang Q., Zhu Y., Yang J., Fu Q. (2021). Dual-atom Pt heterogeneous catalyst with excellent catalytic performances for the selective hydrogenation and epoxidation. Nat. Commun..

[20] Tong M., Sun F., Xie Y., Wang Y., Yang Y., Tian C., Wang L., Fu H. (2021). Operando Cooperated Catalytic Mechanism of Atomically Dispersed Cu-N-4 and Zn-N-4 for Promoting Oxygen Reduction Reaction. Angew. Chem. Int. Ed..

[21] Wan W., Zhao Y., Wei S., Triana C.A., Li J., Arcifa A., Allen C.S., Cao R., Patzke G.R. (2021). Mechanistic insight into the active centers of single/dual-atom Ni/Fe-based oxygen electrocatalysts. Nat. Commun..

[22] Hou C.-C., Wang H.-F., Li C., Xu Q. (2020). From metal-organic frameworks to single/dual-atom and cluster metal catalysts for energy applications. Energy Environ. Sci..

[23] Zhang S., Wu Y., Zhang Y.-X., Niu Z. (2021). Dual-atom catalysts: Controlled synthesis and electrocatalytic applications. Sci. China-Chem..

[24] Wang C., Ying J., Zhang X., Zhang B., Tian A.-X., Wang X.-L. (2021). Multifunctional photoelectric sensors and catalysts for CO_2_RR and Cr(VI) solution based on a series of POM-based materials. CrystEngComm.

[25] Yin F., Lin X., He X., Chen B., Li G., Yin H. (2019). High faraday efficiency for electrochemical nitrogen reduction reaction on Co@N-doped carbon derived from a metal-organic framework under ambient conditions. Mater. Lett..

[26] Liu X., Li X., An M., Gao Y., Cao Z., Liu J. (2020). W–N/C@Co9S8@WS2-hollow carbon nanocage as multifunctional electrocatalysts for DSSCS, ORR and OER. Electrochim. Acta.

[27] Zhong X., Ye S., Tang J., Zhu Y., Wu D., Gu M., Pan H., Xu B. (2021). Engineering Pt and Fe dual-metal single atoms anchored on nitrogen-doped carbon with high activity and durability towards oxygen reduction reaction for zinc-air battery. Appl. Catal. B Environ..

[28] Wang C., Li Y., Zheng L., Zhang C., Wang Y., Shan W., Liu F., He H. (2021). A nonoxide catalyst system study: Alkali metal-promoted Pt/AC catalyst for formaldehyde oxidation at ambient temperature. ACS Catal..

[29] Tian M.Z., Liu S.J., Wang L.L., Ding H., Zhao D., Wang Y.Q., Cui J.H., Fu J.F., Shang J., Li G.K. (2020). Complete degradation of gaseous methanol over Pt/FeO_x_ catalysts by normal temperature catalytic ozonation. Environ. Sci. Technol..

[30] Pei J.J., Wang T., Sui R., Zhang X.J., Zhou D.N., Qin F.J., Zhao X., Liu Q.H., Yan W.S., Dong J.C. (2021). N-Bridged Co-N-Ni: New bimetallic sites for promoting electrochemical CO_2_ reduction. Energy Environ. Sci..

[31] Feng S.Q., Lin X.S., Song X.G., Mei B.B., Mu J.L., Li J.W., Liu Y., Jiang Z., Ding Y.J. (2021). Constructing efficient single Rh sites on activated carbon via surface carbonyl groups for methanol carbonylation. ACS Catal..

[32] Wang F., Miao Z., Mu J., Zhao Y., Liang M., Meng J., Wu X., Zhou P., Zhao J., Zhuo S. (2022). A Ni nanoparticles encapsulated in N-doped carbon catalyst for efficient electroreduction CO_2_: Identification of active sites for adsorption and activation of CO_2_ molecules. Chem. Eng. J..

[33] Gao J., Hou Z.Y., Liu X.S., Zeng Y.W., Luo M.F., Zheng X.M. (2009). Methane autothermal reforming with CO_2_ and O_2_ to synthesis gas at the boundary between Ni and ZrO_2_. Int. J. Hydrogen Energy.

[34] Yao P., Zhang J., Qiu Y., Zheng Q., Zhang H., Yan J., Li X. (2021). Atomic-dispersed coordinated unsaturated nickel-nitrogen sites in hollow carbon spheres for the efficient electrochemical CO_2_ reduction. ACS Sustain. Chem. Eng..

[35] Feng Y., Long S., Chen B., Jia W., Xie S., Sun Y., Tang X., Yang S., Zeng X., Lin L. (2021). Inducing electron dissipation of pyridinic N enabled by single Ni–N_4_ sites for the reduction of aldehydes/ketones with ethanol. ACS Catal..

[36] Yu J., Li J., Xu C.-Y., Liu Q., Liu J., Chen R., Zhu J., Li R., Wang J. (2021). Atomically dispersed Ni–N_4_ species and Ni nanoparticles constructing N-doped porous carbon fibers for accelerating hydrogen evolution. Carbon.

[37] Wei B., Wu W., Xie D., Nastasi M., Wang J. (2021). Strength, plasticity, thermal stability and strain rate sensitivity of nanograined nickel with amorphous ceramic grain boundaries. Acta Mater..

[38] Mao F., Liu P.F., Yang P., Gu J., Yang H.G. (2020). One-step coating of commercial Ni nanoparticles with a Ni, N-codoped carbon shell towards efficient electrocatalysts for CO_2_ reduction. Chem. Commun..

[39] Yu P., Luo Z., Wang Q., Fang M., Zhou J., Wang W., Liang X., Cai W. (2020). Activated carbon-based CO_2_ uptake evaluation at different temperatures: The correlation analysis and coupling effects of the preparation conditions. J. CO_2_ Util..

[40] Dong N., Chen M., Ye Q., Zhang D., Dai H. (2021). Catalytic elimination of carbon monoxide, ethyl acetate, and toluene over the Ni/OMS-2 Catalysts. Catalysts.

[41] Hashem A., Fletcher A.J., Younis H., Mauof H., Abou-Okeil A. (2020). Adsorption of Pb(II) ions from contaminated water by 1,2,3,4-butanetetracarboxylic acid-modified microcrystalline cellulose: Isotherms, kinetics, and thermodynamic studies. Int. J. Biol. Macromol..

[42] Zhao Y., Chen D., Liu J., He D., Cao X., Han C., Lu J., Luo Y. (2020). Tuning the metal-support interaction on chromium-based catalysts for catalytically eliminate methyl mercaptan: Anchored active chromium species through surface hydroxyl groups. Chem. Eng. J..

[43] Nabais J., Carrott P., Carrott M., Menéndez J. (2004). Preparation and modification of activated carbon fibres by microwave heating. Carbon.

[44] Jiang W., Li Y., Xu Y., Jiang T., Zhao M., Deng M., Wu R., Wang Y. (2021). Carbon nanotube-bridged N-doped mesoporous carbon nanosphere with atomic and nanoscaled M (M = Fe, Co) species for synergistically enhanced oxygen reduction reaction. Chem. Eng. J..

[45] Xu D., Pan Y., Zhu L., Yusran Y., Zhang D., Fang Q., Xue M., Qiu S. (2017). Simple coordination complex-derived Ni NP anchored N-doped porous carbons with high performance for reduction of nitroarenes. Crystengcomm.

[46] Liang S., Jiang Q., Wang Q., Liu Y. (2021). Revealing the real role of nickel decorated nitrogen-doped carbon catalysts for electrochemical reduction of CO_2_ to CO. Adv. Energy Mater..

[47] Wang X., Sang X., Dong C.-L., Yao S., Shuai L., Lu J., Yang B., Li Z., Lei L., Qiu M. (2021). Proton capture strategy for enhancing electrochemical CO_2_ reduction on atomically dispersed metal-nitrogen active sites. Angew. Chem. Int. Ed..

[48] Mamtani K., Jain D., Zemlyanov D., Celik G., Luthman J., Renkes G., Co A.C., Ozkan U.S. (2016). Probing the Oxygen Reduction Reaction Active Sites over Nitrogen-Doped Carbon Nanostructures (CN) in Acidic Media Using Phosphate Anion. ACS Catal..

[49] Yang J., Zeng D., Zhang Q., Cui R., Hassan M., Dong L., Li J., He Y. (2020). Single Mn atom anchored on N-doped porous carbon as highly efficient fenton-like catalyst for the degradation of organic contaminants. Appl. Catal. B Environ..

[50] Xiang N., Tian J., Li Q., Hou Y., Huang Z. (2024). Promotional mechanism of nitrogen-doping in activated carbon for formaldehyde removal: Enhanced attractive noncovalent interactions coupled with Cannizzaro-type disproportionation reaction. Sep. Purif. Technol..

[51] Bai P., Tian F., Wang H., Yang T., Bi X., Chai Z., Wang X. (2019). Electrocatalytic enhancement of 0D/1D/2D multidimensional PtCo alloy@cobalt benzoate/graphene composite catalyst for alcohol electro-oxidation. Adv. Mater. Interfaces.

[52] Ma M., Yang R., Jiang Z., Chen C., Liu Q., Albilali R., He C. (2021). Fabricating M/Al_2_O_3_/cordierite (M = Cr, Mn, Fe, Co, Ni and Cu) monolithic catalysts for ethyl acetate efficient oxidation: Unveiling the role of water vapor and reaction mechanism. Fuel.

[53] Si W., Wang Y., Zhao S., Hu F., Li J. (2016). A facile method for in situ preparation of the MnO_2_/LaMnO_3_ catalyst for the removal of toluene. Environ. Sci. Technol..

[54] Luo Z., Fang Y., Zhou M., Wang X. (2019). A borocarbonitride ceramic aerogel for photoredox catalysis. Angew. Chem. Int. Ed..

[55] Li X., Lin B., Li H., Yu Q., Ge Y., Jin X., Liu X., Zhou Y., Xiao J. (2018). Carbon doped hexagonal BN as a highly efficient metal-free base catalyst for Knoevenagel condensation reaction. Appl. Catal. B Environ..

[56] Lv L., Tang B., Ji Q., Li N., Wang Y., Feng S., Duan H., Wang C., Tan H., Yan W. (2023). Highly exposed NiFeO_x_ nanoclusters supported on boron doped carbon nanotubes for electrocatalytic oxygen evolution reaction. Chin. Chem. Lett..

[57] Xu Y., Zhang W., Li Y., Lu P., Wu Z.-S. (2020). A general bimetal-ion adsorption strategy to prepare nickel single atom catalysts anchored on graphene for efficient oxygen evolution reaction. J. Energy Chem..

[58] Ma L., Wang D., Li J., Bai B., Fu L., Li Y. (2014). Ag/CeO_2_ nanospheres: Efficient catalysts for formaldehyde oxidation. Appl. Catal. B Environ..

[59] Zhang N., Zhang X., Tao L., Jiang P., Ye C., Lin R., Huang Z., Li A., Pang D., Yan H. (2021). Silver single-atom catalyst for efficient electrochemical CO_2_ reduction synthesized from thermal transformation and surface reconstruction. Angew. Chem. Int. Ed..

[60] Romand M., Roubin M., Deloume J.P. (1978). ESCA studies of some copper and silver selenides. J. Electron. Spectrosc. Relat. Phenom..

[61] Schön G., Tummavuori J., Lindström B., Enzell C., Swahn C.-G. (1973). ESCA studies of Ag, Ag_2_O and AgO. Acta Chem. Scand..

[62] Liu R., Wu H., Shi J., Xu X., Zhao D., Ng Y.H., Zhang M., Liu S., Ding H. (2022). Recent progress on catalysts for catalytic oxidation of volatile organic compounds: A review. Catal. Sci. Technol..

[63] Chen W.R., Sharpless C.M., Linden K.G., Suffet I.H. (2006). Treatment of volatile organic chemicals on the EPA contaminant candidate list using ozonation and the O_3_/H_2_O_2_ advanced oxidation process. Environ. Sci. Technol..

[64] Zhang G., Xie M., Zhao J., Wei S., Zheng H., Zhang S. (2021). Key structural features that determine the selectivity of UV/acetylacetone for the degradation of aromatic pollutants when compared to UV/H_2_O_2_. Water Res..

[65] Hansen A.S., Bhagde T., Moore K.B., Moberg D.R., Jasper A.W., Georgievskii Y., Vansco M.F., Klippenstein S.J., Lester M.I. (2021). Watching a hydroperoxyalkyl radical (QOOH) dissociate. Science.

[66] Lao Y., Jiang X., Huang J., Zhang Z., Wang X. (2020). Catalytic oxidation of ethyl acetate on Ce-Mn-O catalysts modified by La. Rare Met..

[67] Yang Y., Li Y., Zhang Q., Zeng M., Wu S., Lan L., Zhao X. (2018). Novel photoactivation and solar-light-driven thermocatalysis on ε-MnO_2_ nanosheets lead to highly efficient catalytic abatement of ethyl acetate without acetaldehyde as unfavorable by-product. J. Mater. Chem. A.

