# Supplementary material for: Catalytic Oxidation of Benzene over Atomic Active Site AgNi/BCN Catalysts at Room Temperature
Source: Molecules. 2024 Mar 25;29(7):1463. doi: 10.3390/molecules29071463 (PMC11013234; doi:10.3390/molecules29071463)
Supplement: Supplementary file 1 [file molecules-29-01463-s001.zip › molecules-2915486-supplementary.pdf]

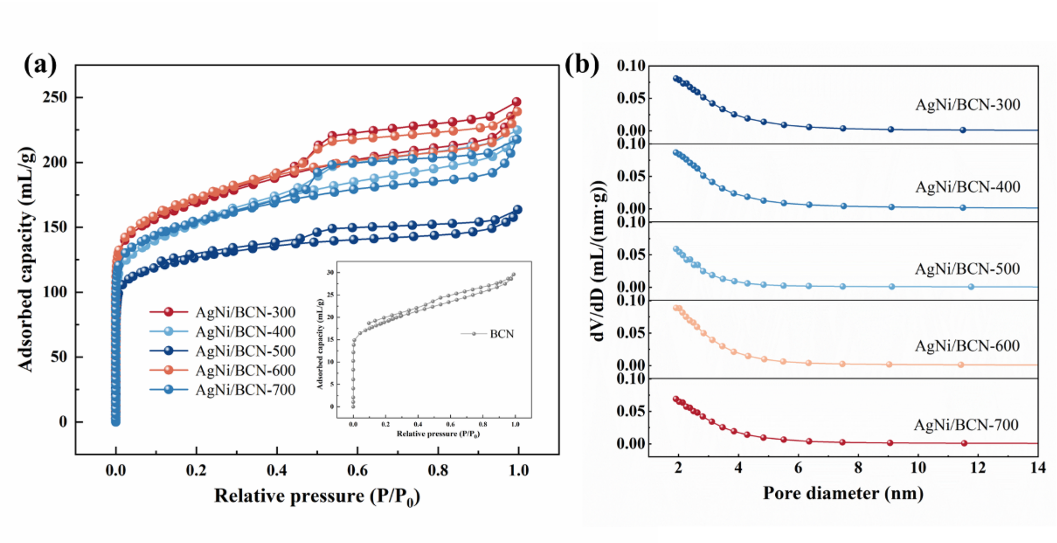

Figure S1 N_2 adsorption-desorption experiments: (a) N_2 adsorption-desorption isotherms; (b) Pore size distribution.

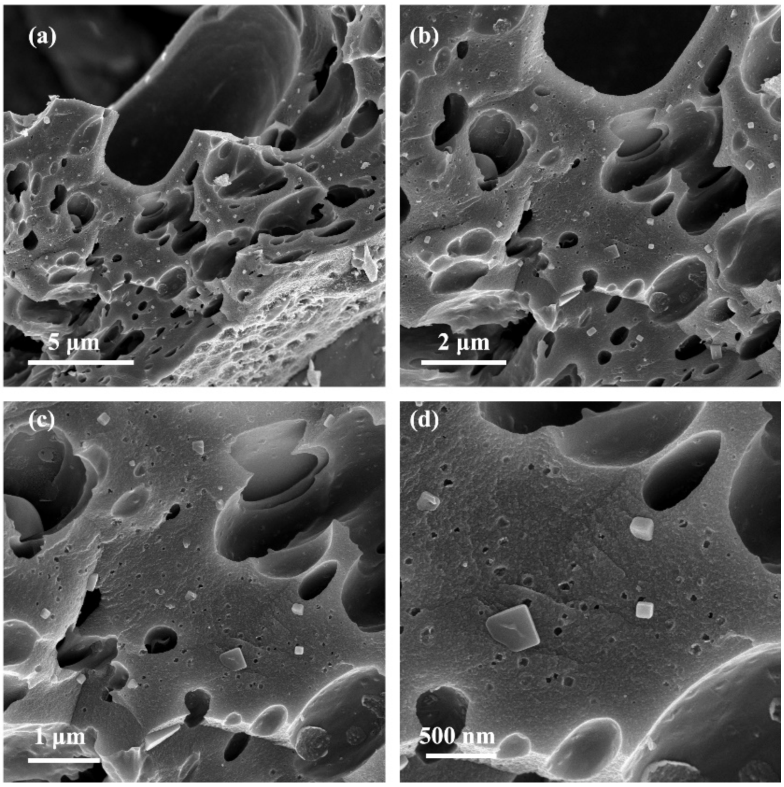

Figure S2 SEM images of BCN aerogel carrier.

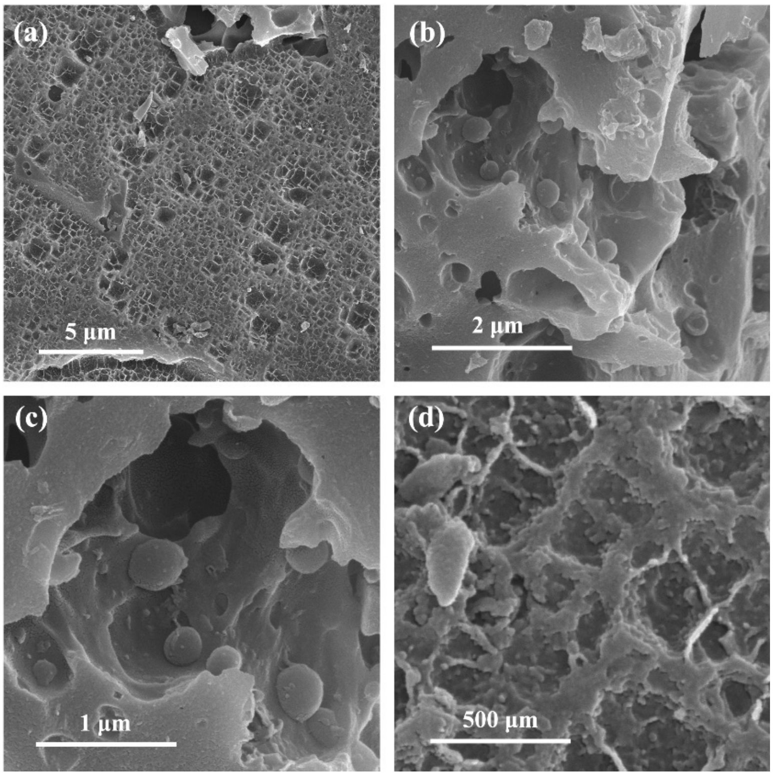

Figure S3 SEM images of AgNi/BCN-400 catalyst.

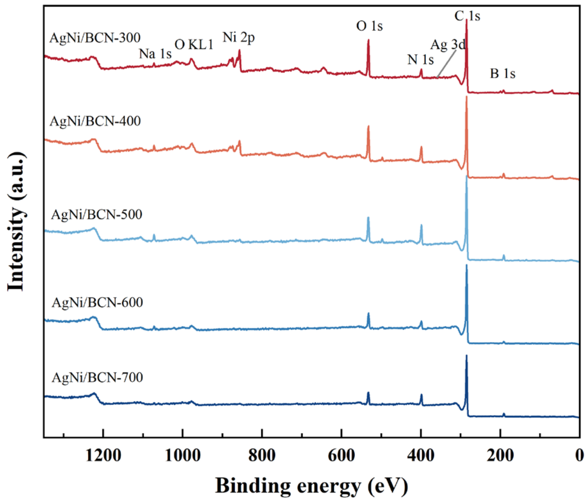

Figure S4 XPS survey scan of AgNi/BCN-X catalysts.

Table. S1 Experimental agents

Reagent	Concentration	Reagent	Concentration
C_6H_6	AR（≥99.5%）	$Na_2CO_3·10H_2O$	98%
$(C_6H_{10}O_5)_n$	BR	$H_2PtCl_6·6H_2O$	AR
CH_4N_2O	AR（≥99.5%）	$AuCl_4H$	97%
H_3BO_3	99.5%	$C_{14}H_{10}Fe_2O_4$	>96.0%(T)
$Ni(NO_3)_2·6H_2O$	AR（≥98.0%）	N_2	99.999%
NaCl	AR	He	5N
$AgNO_3$	AR（≥99.0%）	H_2/N_2	5N
HNO_3	AR	N_2	5N

Table. S2 Relative content of oxygen species in AgNi/BCN-X catalysts.

Samples	O_{latt}	O_{ads}	Adsorbed water species	O_{ads}/O_{latt}
AgNi/BCN-300	4.17	39.49	56.33	9.45
AgNi/BCN-400	4.30	42.29	53.42	9.84
AgNi/BCN-500	4.61	25.62	69.77	5.56
AgNi/BCN-600	5.92	40.14	53.93	6.78
AgNi/BCN-700	11.34	35.56	53.10	3.14

Table. S3 Relative content of boron species in AgNi/BCN-X catalysts.

Samples	B-C	B-N	B-O
AgNi/BCN-300	11.11	44.44	44.44
AgNi/BCN-400	12.74	51.38	35.88
AgNi/BCN-500	11.14	42.71	46.14
AgNi/BCN-600	12.16	78.83	9.01
AgNi/BCN-700	12.02	73.01	14.96
